# Janus kinases inhibitors for coronavirus disease-2019: A pairwise and Bayesian network meta-analysis

**DOI:** 10.3389/fmed.2022.973688

**Published:** 2022-11-23

**Authors:** Jianyi Niu, Zhiwei Lin, Zhenfeng He, Xiaojing Yang, Lijie Qin, Shengchuan Feng, Lili Guan, Luqian Zhou, Rongchang Chen

**Affiliations:** ^1^State Key Laboratory of Respiratory Disease, National Clinical Research Center for Respiratory Disease, Guangzhou Institute of Respiratory Health, First Affiliated Hospital of Guangzhou Medical University, Guangzhou, China; ^2^Respiratory Mechanics Laboratory, Guangzhou Institute of Respiratory Health, First Affiliated Hospital of Guangzhou Medical University, Guangzhou, China; ^3^Key Laboratory of Shenzhen Respiratory Diseases, Institute of Shenzhen Respiratory Diseases, Shenzhen People’s Hospital (The First Affiliated Hospital of Southern University of Science and Technology, The Second Clinical Medical College of Jinan University), Shenzhen, Guangdong, China

**Keywords:** COVID-19, JAK inhibitors, mortality, adverse events, network meta-analysis

## Abstract

**Background:**

JAK (Janus kinases) inhibitors have been proposed as a promising treatment option for the coronavirus disease-2019 (COVID-19). However, the benefits of JAK inhibitors and the optimum thereof for COVID-19 have not been adequately defined.

**Methods:**

Databases were searched from their inception dates to 17 June 2022. Eligible studies included randomized controlled trials and observational studies. Extracted data were analyzed by pairwise and network meta-analysis. The primary outcome was the coefficient of mortality.

**Results:**

Twenty-eight studies of 8,206 patients were included and assessed qualitatively (modified Jadad and Newcastle–Ottawa Scale scores). A pairwise meta-analysis revealed that JAK inhibitors effectively reduced the mortality (OR = 0.54; 95% CI: 0.46–0.63; *P* < 0.00001; *I*^2^ = 32%) without increasing the risk of adverse events (OR = 1.02; 95% CI: 0.88–1.18; *P* = 0.79; *I*^2^ = 12%). In a network meta-analysis, clinical efficacy benefits were seen among different types of JAK inhibitors (baricitinib, ruxolitinib, and tofacitinib) without the observation of a declined incidence of adverse events. The assessment of rank probabilities indicated that ruxolitinib presented the greatest likelihood of benefits regarding mortality and adverse events.

**Conclusion:**

JAK inhibitors appear to be a promising treatment for COVID-19 concerning reducing mortality, and they do not increase the risk of adverse events vs. standard of care. A network meta-analysis suggests that mortality benefits are associated with specific JAK inhibitors, and among these, ruxolitinib presents the greatest likelihood of having benefits for mortality and adverse events.

**Systematic review registration:**

[www.crd.york.ac.uk/prospero], identifier [CRD42022343338].

## Background

The severe acute respiratory syndrome-coronavirus-2 [SARS-CoV-2/coronavirus disease-2019 (COVID-19)] pandemic has emerged as an extraordinary challenge to public health. According to the World Health Organization’s most recent weekly epidemiological update on COVID-19 (17 June 2022), the cumulative number of cases reported globally since 2019 exceeds 535 million, and the number of deaths caused by this infection has surpassed 6.31 million. The number of people diagnosed has been increasing globally as variants continue to emerge, even as vaccines have been administered in multiple countries.

The primary cause of death from COVID-19 is acute respiratory distress syndrome, while cytokine release syndrome (CRS), characterized by increased interleukin (IL)-6, IL-2, IL-7, IL-10, is thought to be the main reason for multiple organ failure (1). Therefore, treatment of cytokine storm has been proposed as a key part of rescuing severe COVID-19 disease. Several cytokines associated with COVID-19 disease employ a unique intracellular signaling pathway mediated by Janus kinase (JAK). JAK inhibition provides an attractive therapeutic strategy for CRS (2). Accordingly, effective inhibition of cytokine storms is crucial for preventing severe COVID-19 complications and reducing mortality (3).

JAK-signal transducer and activator of transcription (STAT) signaling is critical to multiple cellular processes, including survival, differentiation, and proliferation (4). Over the past decade, JAK inhibitors have known a wide range of applications in the clinic and have been constantly designed for new molecules. Ruxolitinib, one of the oldest JAK inhibitors, is the drug most commonly used in patients with hematologic disorders, while other JAK inhibitors such as baricitinib and tofacitinib are more commonly used in systemic rheumatic diseases (5). JAK-STAT inhibitors can block many proinflammatory and anti-inflammatory cytokines and have good pharmacodynamics. At the same time because of its short half-life, the extent and duration of cytokine arrest can be easily monitored by adjusting the dose and duration of treatment (6, 7).

Four previous meta-analyses have reported that JAK inhibitors could be beneficial in the treatment of COVID-19 (8–11). However, all of these studies were pairwise meta-analyses comparing the JAK inhibitors with the standard of care (SOC). There was no comparison of efficacy and safety between JAK inhibitors in COVID-19 patients. To solve this problem, this research sought to further explore the efficacy and safety of JAK inhibitors in patients with COVID-19 by updating the latest relevant evidence and using a network meta-analysis (NMA) approach to assess the efficacy and safety of three JAK inhibitors (baricitinib, ruxolitinib and tofacitinib) through indirect comparison. The primary outcome was mortality. Because many symptoms of COVID-19 overlap with the adverse events of JAK inhibitors, in all relevant cohort studies, it has not been clarified whether the adverse events were caused by symptoms of COVID-19 disease itself or by taking JAK inhibitors (12, 13). Therefore, this study only focused on whether treatment of JAK inhibitors increased the overall risk of these adverse events compared with SOC, and the adverse events analyzed in this study were based on the data of the included studies.

## Methods

This systematic review and meta-analysis were performed under the instruction of PRISMA guidance,^1^ and the protocol for research was registered at PROSPERO (CRD42022343338).

### Search strategy

This was a pairwise and Bayesian network meta-analysis study of clinical trials and observational studies. Published studies included in the meta-analysis were those that fit within the following PICO framework (P: Populations, hospitalized COVID-19 patients; I: Interventions, treatment with JAK inhibitors; C: Comparator/Control, a group of patients who only received SOC therapy and was not treated with JAK inhibitors; O: Outcomes, mortality and the risk of adverse events), and met the Preferred Reporting Items for pairwise and Bayesian network meta-analysis (PRISMA) criteria (14).

PubMed, Embase, Clinical Trial, and Web of Science were each searched from their inception to 17 June 2022 by three independent reviewers. The keywords “JAK inhibitors OR Baricitinib OR Ruxolitinib OR Tofacitinib” AND “SARS-CoV-2 OR coronavirus disease 2019 OR COVID-19” were used. The investigators independently screened titles and abstracts generated by the search. The search was reworked before the final analyses to review the latest studies.

### Inclusion and exclusion criteria

The comparative trials that met all the following criteria was included: (1) The studies were in English; (2) a JAK inhibitor (baricitinib, ruxolitinib, tofacitinib or nezulcitinib) was used alone or with other therapies in the experimental group who were suffering from COVID-19, while the SOC was applied in the control group. SOC in the control groups included patients with the application of oxygen *via* low-flow or high-flow devices, invasive or non-invasive ventilation and other medication like steroids or remdesivir; (3) the survey was conducted among adults; (4) Clinical outcomes of interest (all-cause mortality and adverse events) were reported; (5) the included researches were cohort, randomized or non-randomized clinical trial research; (6) the included researches were correspondence or review articles, case-series or case report studies.

### Data extraction and study quality assessment

After selection, the full text articles in electronic versions were then carefully evaluated for data extraction. Two independent reviewers extracted data independently using predefined standardized forms and one independent reviewer joined when any disagreements existed. Each full article that met the inclusion criteria was carefully reviewed, and the following baseline information was extracted: first author, publication year, study type, basic information of the participants (age, severity), the total number of participants, number of participants receiving JAK inhibitors (baricitinib, ruxolitinib, tofacitinib and nezulcitinib), number of participants receiving SOC, the regimen of JAK inhibitors or SOC and the modified Jadad (15) or Newcastle–Ottawa Scale (NOS) score (16). The outcome measures were mortality and the risk of adverse events.

Three independent reviewers independently assessed the quality of each study involved in this review. Randomized clinical trials (RCTs) and clinical trials included in the final analyses were scored by one independent reviewer to formally assess the risk of bias using the modified Jadad score. Observational studies included in the final analyses were scored by one independent reviewer using the NOS score. There was adjudication by one independent reviewer when there was disagreement.

The modified Jadad scale was used to evaluate the quality of RCTs and clinical trials, including the presence of randomization (0 or 2), blinding (0 or 2), description of withdrawals and dropouts (0 or 1), inclusion/exclusion criteria (0 or 1), adverse effects (0 or 1) and statistical analysis (0 or 1) for each study. The studies were scored from zero to eight, with 1–3 signifying low quality and 4–8 signifying high quality (17). The NOS score was used to evaluate the quality of observational studies. The representativeness of the exposed cohort (0 or 1), selection of the non-exposed cohort (0 or 1), ascertainment of exposure (0 or 1), whether the subjects had the disease they were studying at the start of the study (0 or 1), comparability (0 or 1), non-comparability (0 or 1), method (0 or 1), follow-up time (0 or 1) and adequacy of follow-up of cohorts (0 or 1) were reported for the NOS score. Research is graded as good quality if it scores ≥ 7 (18).

### Statistical analysis

Review Manager V.5.3 software (Cochrane Collaboration, Oxford, UK) for pairwise meta-analysis was used. To calculate the odds ratio (OR) and its 95% confidence interval (95% CI) for dichotomous outcomes (mortality and adverse events), the Mantel-Haenszel formula was utilized. Heterogeneity was assessed using the Cochran Q statistic and the I^2^ statistic. The I^2^ values ≤ 50% were considered an acceptable heterogeneity between studies, and the fixed-effects model was selected. Otherwise, determine the source of heterogeneity through subgroup analysis, and conduct sensitivity analysis or use random effect model. For subgroup analyses, the studies were split into four subgroups according to different drugs and applied a pairwise meta-analysis model separately in each subgroup.

The R software (version 3.6.1) and R package “gemtc” were mainly employed to construct the Bayesian network meta-analysis with 3 chains simulated for 50,000 iterations using The Markov Chain Monte Carlo method (17). Fixed-effect model was utilized, and the random-effect model was applied when the heterogeneity was high. The Bayesian approach also provided overall ranking probabilities for each treatment, making it possible to rank each outcome measurement from the best to the worst, and were then visualized by rankogram. The drug with the largest proportion of dark gray rectangles (best treatment) represents the drug that is considered to have the highest probability of the most negative value of mortality or adverse events.

## Results

### Selection and characteristics of the studies

**FIGURE 1:**
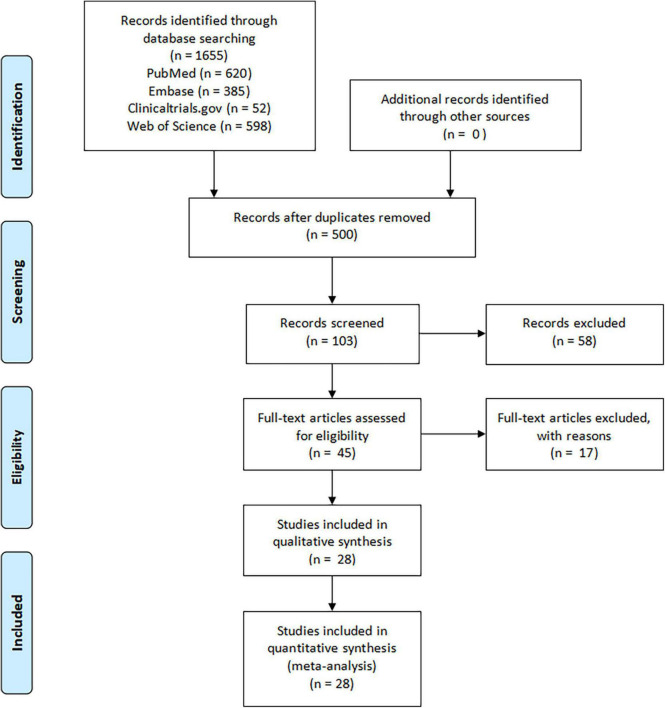
The PRISMA flow diagram.

**FIGURE 2:**
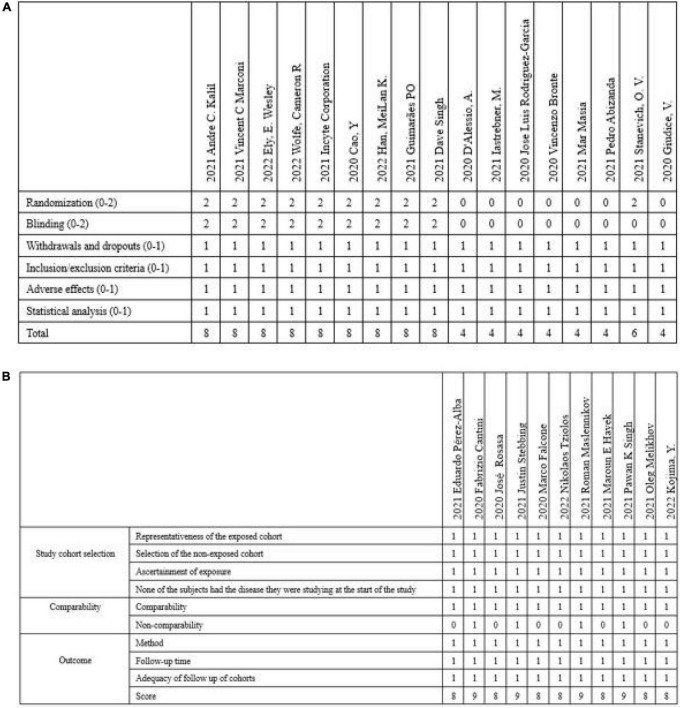
The quality assessment for these studies included in the article. **(A)** Modified Jadad score was used to score RCTs and clinical trials included. **(B)** NOS score was used to score observational studies. RCTs, randomized clinical trials; NOS, Newcastle–Ottawa Scale.

The PRISMA flow diagram was shown in Figure 1. A total of 3,310 articles were identified initially. 103 highly relevant articles were identified by searching titles and abstracts and eliminating repetitions. After examining the content further, 28 studies (19–46) comprising 8,826 patients remained. Of the 28 studies, nine were double-blind, RCTs, two were non-randomized clinical trials, and 17 were prospective cohort or retrospective cohort studies (Table 1). The quality assessment for these studies was shown in Figure 2.

**TABLE 1:** Characteristics of studies included in the meta-analysis.

References	Sample size	Design	Overall age mean ± SD	Outcome	Type of JAK—inhibitor	Regimen of JAK—inhibitor	Regimen of standard of care	Patient category	JAK—inhibitor vs. control *n* (%)
Kalil et al. (19)	1,033	Double-blind randomized clinical trial	55.4 ± 15.7	(1) Mortality (2) Safety	Baricitinib	4 mg/day for 2 weeks	Placebo	Moderate to severe	515 (49.8%) vs. 518 (50.2%)
Marconi et al. (20)	1,525	Double-blind Randomized clinical trial	57.6 ± 14.1	(1) Mortality (2) Safety	Baricitinib	4 mg/day for 2 weeks	Placebo + standard of care	Moderate to severe	764 (50.1%) vs. 761 (49.9%)
Ely et al. (45)	101	Double-blind randomized clinical trial	58.598 ± 13.788	(1) Mortality (2) Safety	Baricitinib	4 mg/day for up to 14 days or until hospital discharge	Placebo + standard of care	Severe	51 (50.5%) vs. 50 (49.5%)
Wolfe et al. (43)	1,010	Double-blind randomized clinical trial	58.3 ± 14.0	(1) Mortality (2) Safety	Baricitinib + remdesivir	4 mg/day for up to 14 days or until hospital discharge	Dexamethasone + remdesivir + placebo	Moderate to severe	516 (51.1%) vs. 494 (48.9%)
Incyte Corporation (NCT04377620) et al. (40)	211	Double-blind Randomized clinical trial	63.4 ± 12.69	(1) Mortality (2) Safety	Ruxolitinib	5 mg or 1 mg twice daily	placebo + standard of care	Moderate to severe	164(77.7%) vs. 47 (22.3%)
Cao et al. (21)	41	Single-blind Randomized clinical trial	63 ± 7.4	(1) Mortality (2) Safety	Ruxolitinib	5 mg, twice daily for 2 weeks	Placebo (Vitamin C) + standard of care	Severe	20 (48.8%) vs. 21 (51.2%)
Han et al. (44)	432	Double-blind randomized clinical trial	56.5 ± 13.3	(1) Mortality (2) Safety	Ruxolitinib	5 mg twice daily	Placebo tablets 5 mg twice per day for 14 days	Moderate to severe	287 (66.4%) vs. 145 (33.6%)
Guimar aes et al. (22)	289	Double-blind randomized clinical trial	56 ± 14	(1) Mortality (2) Safety	Tofacitinib	10-mg twice daily for 14 days or until hospital discharge	Placebo 10 mg twice daily for up to 14 days + standard of care	Moderate to severe	144 (49.8%) vs. 145 (50.2%)
Singh et al. (46)	25	Double-blind randomized clinical trial	57.32 ± 12.86	(1) Mortality	Nezulcitini	1, 3 10 mg daily for up to 7 days	Placebo inhalation	Moderate to severe	19 (76%) vs. 6 (23%)
D’Alessio et al. (27)	75	Non-randomized clinical trial	67.6 ± 5.1	(1) Mortality	Ruxolitinib	5 mg, twice daily for 7 days, then tapered to 5 mg/day for a total of 10 days	Hydroxychloroquine + lopinavir/ritonavir	Severe	32 (42.6%) vs. 43 (57.4%)
Iastrebner et al. (28)	102	Clinical trial	57.26 ± 11.41	(1) Mortality (2) Safety	Ruxolitinib	5 mg twice daily	Standard of care	Severe	51 (50.0%) vs. 51 (50.0%)
Rodriguez-Garcia et al. (23)	387	Prospective cohort	62.3 ± 14.8	(1) Mortality (2) Safety	Baricitinib	4 mg/day for 5–10 days	Standard of care + corticosteroids	Moderate to severe	117 (30.2%) vs. 270 (69.8%)
Bronte et al. (24)	76	Prospective cohort	73.5 ± 13.8	(1) Mortality	Baricitinib	4 mg, twice daily for 2 days, followed by 4 mg/day for the remaining 7 days	Hydroxychloroquine + lopinavir/ritonavir + supportive care	Severe	20 (26.3%) vs. 56 (73.7%)
Masia et al. (25)	190	Prospective cohort	60.11 ± 3.41	(1) Mortality (2) Safety	Baricitinib	Not available	Standard of care (tocilizumab + corticosteroids)	Severe	95 (50%) vs. 95 (50%)
Abizanda et al. (26)	156	Prospective cohort	68.5 ± 12.4	(1) Mortality	Baricitinib	Not available	Standard of care	Moderate to severe	78 (50%) vs. 78 (50%)
Stanevich et al. (29)	292	Prospective cohort	58.1 ± 13.3	(1) Mortality (2) Safety	Ruxolitinib	5–10 mg/day until oxygen support withdrawal	Dexamethasone for 5– 10 days	Severe	146 (50%) vs. 146 (50%)
Giudice et al. (30)	17	Prospective cohort	63.5 ± 12.5	(1) Mortality (2) Safety	Ruxolitinib	10 mg, twice daily for 14 days	Hydroxychloroquine + supportive care	Severe	7 (41.1%) vs. 10 (58.9%)
Pérez-Alba et al. (32)	197	Retrospective cohort	59.9 ± 14.9	(1) Mortality (2) Safety	Baricitinib	4 mg/day for 2 weeks	Dexamethasone 6 mg/day for 10 days	Moderate to severe	123 (62.4%) vs. 74 (37.6%)
Cantini et al. (34)	191	Retrospective cohort	67 ± 14	(1) Mortality (2) Safety	Baricitinib	4 mg/day for 2 weeks	Hydroxychloroquine + lopinavir/ritonavir	Mild to moderate	113 (59.1%) vs. 78 (40.9%)
Rosas et al. (33)	29	Retrospective cohort	67.8 ± 13.6	(1) Mortality	Baricitinib	4 mg/day for 2 weeks	Standard of care	Moderate to severe	12 (41.3%) vs. 17 (58.7%)
Stebbing et al. (35)	179	Prospective cohort	66 ± 26.6	(1) Mortality (2) Safety	Baricitinib	4 mg/day for 2 weeks	Standard of care	Moderate to severe	37 (20.6%) vs. 142 (79.4%)
Falcone et al. (37)	278	Retrospective cohort	69.92 ± 3.99	(1) Mortality	Baricitinib	Not available	Standard of care	Moderate to severe	40 (14.4%) vs. 238 (85.6%)
Tziolos et al. (36)	369	Retrospective cohort	65.2 ± 13.6	(1) Mortality	Baricitinib	4 mg once a day for up to 14 days or until hospital discharge	Standard of care	severe	193 (52.3%) vs. 176 (47.7%)
Maslennikov et al. (31)	62	Retrospective cohort	64.3 ± 12.5	(1) Mortality (2) Safety	Tofacitinib	10 mg, twice daily on the first day, then 5 mg, twice daily for 4 days	Standard of care	Moderate to severe	32 (51.6%) vs. 30 (48.4%)
Hayek et al. (38)	269	Retrospective cohort	63.92 ± 14.23	(1) Mortality (2) Safety	Tofacitinib	10-mg twice daily for 5 days	Dexamethasone	severe	138 (51.3%) vs. 131 (48.7%)
Singh et al. (39)	50	Retrospective cohort	46.12 ± 13.49	(1) Mortality	Tofacitinib	10 mg, twice daily	Standard of care	severe	25 (50.0%) vs. 25 (50.0%)
Melikhov et al. (41)	522	Prospective cohort	58.62 ± 12.59	(1) Mortality (2) Safety	Tofacitinib	10 mg for 7–14 days	Baricitinib 4 mg for 7–14 days	Mild to severe	320 (61.3%) vs. 202 (38.7%)
Kojima et al. (42)	98	Retrospective cohort	60.68 ± 3.27	(1) Mortality (2) Safety	Tofacitinib	Not available	Baricitinib	Mild to severe	64 (65.3%) vs. 34 (34.7%)

JAK, inhibitor; Janus kinase inhibitor; SD; standard deviation.

### Baseline characteristics of the included studies

Data on the first author, sample size, study design, age and severity of patients, outcome, the specific regimen of JAK-inhibitor used in the experimental group, and the specific regimen of SOC used in the control group were shown in Table 1.

### Study quality assessment

The modified Jadad scale was used to evaluate the quality of RCTs and clinical trials. And the NOS score was used to evaluate the quality of observational studies (Figure 2).

### Pairwise meta-analysis results

#### Mortality

**FIGURE 3:**
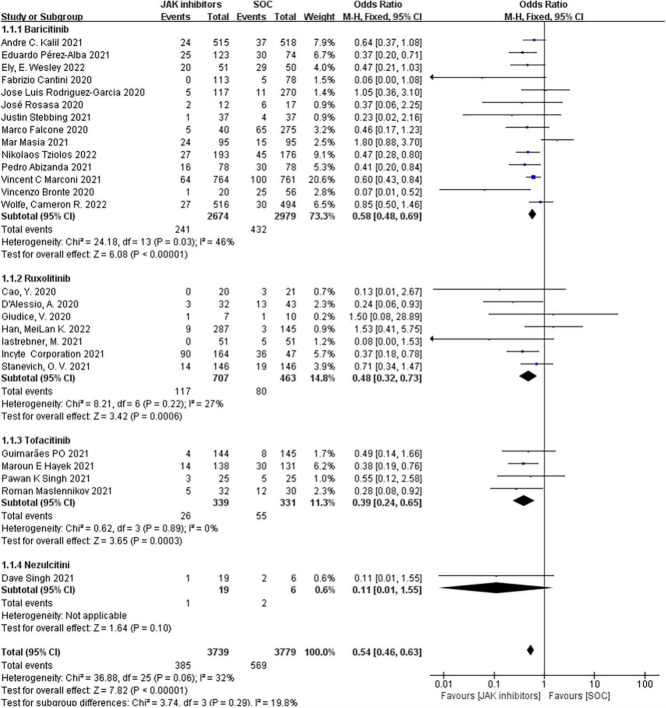
Forest plots of effect of JAK inhibitors compared to SOC on mortality. The study was divided into four subgroups by different drugs. SOC, standard of care.

The outcomes of mortality in the JAK inhibitors groups vs. the SOC groups were shown in Figure 3. Twenty-six studies including 8,206 patients reported the mortality (19–40, 43–47). Pooled results showed that treatment with JAK inhibitors was associated with significantly lower mortality compared with that in the SOC groups (OR = 0.54; 95% CI: 0.46–0.63; *P* < 0.00001; Figure 3). There was low statistical heterogeneity in the fixed effect model (*I*^2^ = 32%).

Our subgroup analyzed by different types of JAK inhibitors found that in the 14 studies of 5,653 patients treated with baricitinib (19, 23–26, 32–37, 43, 45, 47), the effect vs. SOC in patients produced an OR of 0.58 (95% CI: 0.48–0.69; *P* < 0.00001; *I*^2^ = 46%; Figure 3); the seven studies of 1,170 ruxolitinib-treated patients (21, 27–30, 40, 44) reported an OR of 0.48 (95% CI: 0.32–0.73; *P* = 0.0006; *I*^2^ = 27%; Figure 3) vs. SOC; four studies of 670 patients treated with tofacitinib (22, 31, 38, 39) reported an OR of 0.39 (95% CI: 0.24–0.65; *P* = 0.0003; *I*^2^ = 0%; Figure 3) vs. SOC; and only one study of 25 patients treated with nezulcitini (44) reported an OR of 0.11 (95% CI: 0.01–1.55; *P* = 0.10; Figure 3).

#### Adverse events

When examining adverse events associated with JAK inhibitor treatment in patients with COVID-19, the most frequent adverse events were infections, embolisms, liver dysfunction, renal and urinary disorders, and mental disorders. Among these, infection and thrombosis after receiving the drug were the most concerned adverse events for doctors. Therefore, this meta-analysis focused on the selection of the incidence of them during data screening and discussed the comparison with the control.

**FIGURE 4:**
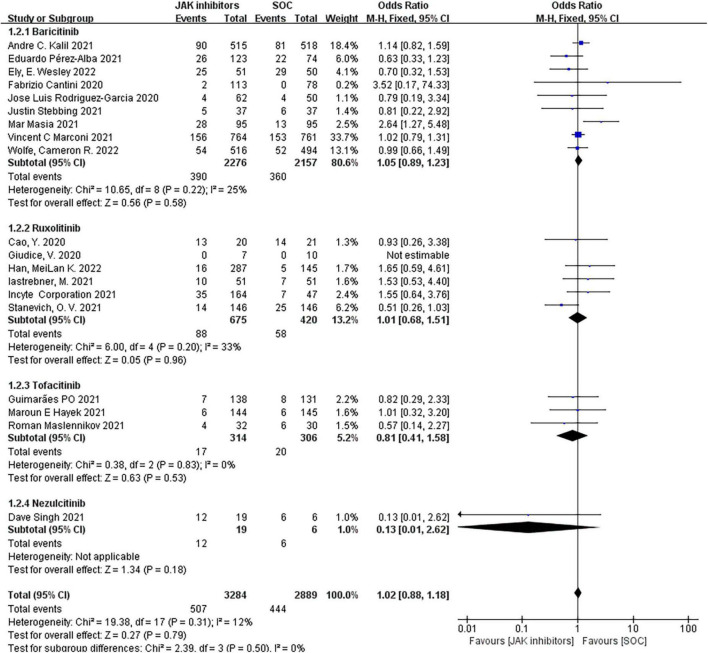
Forest plots of effect of JAK inhibitors compared to SOC on adverse events. The study was divided into four subgroups by different drugs. SOC, standard of care.

Nineteen studies including 6,173 patients investigated the effect of JAK inhibitors on adverse events. The pooled results showed no significant differences between the JAK inhibitors group and the control groups in the overall adverse event (OR = 1.02; 95% CI: 0.88–1.18; *P* = 0.79; *I*^2^ = 12%; Figure 4). The subgroup analyzed by type of JAK inhibitor showed no difference in the adverse event from standard treatment for any of the three drugs.

#### Publication bias and sensitivity analyses

Funnel plot analysis for mortality and the incidence of adverse events presented a relatively symmetrical inverted plot (Supplementary Figure 1), indicating no publication bias. Sensitivity analyses were also conducted to assess the impact of each study on the pooled OR, and the statistical results were not remarkably altered after removing any study (Supplementary Figure 2).

### Network meta-analysis results

**FIGURE 5:**
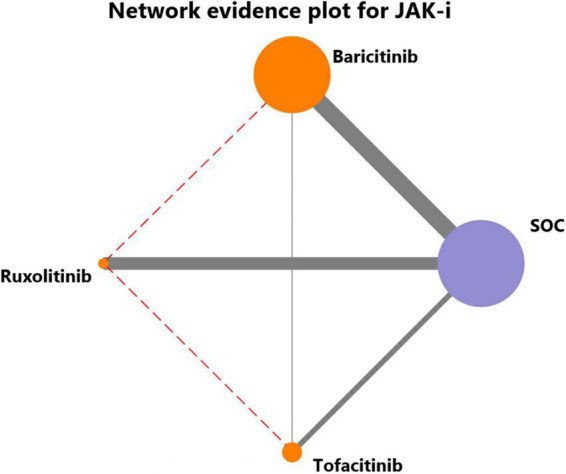
Network evidence for JAK inhibitors to treat COVID-19. The thickness of connecting lines represented the number of trials between each comparator, and the size of each node corresponded to the number of participants who received the same intervention (sample size).

Figure 5 showed a comparison of network evidence from studies of mortality involved in treatment with multiple JAK inhibitors. The thickness of connecting lines represented the number of trials between each comparator, and the size of each node corresponded to the number of participants who received the same intervention (sample size).

#### Mortality

Fifteen trials were comparing the coefficient of mortality among baricitinib-treated patients, seven studies among ruxolitinib-treated patients, and four trials among tofacitinib-treated patients. Thus, this research first pooled direct comparisons to obtain indirect comparisons by comparing baricitinib vs. SOC (19, 23–26, 32–37, 43, 45, 47), ruxolitinib vs. SOC (21, 27–30, 40, 44), tofacitinib vs. SOC (22, 31, 38, 39), and tofacitinib vs. baricitinib (41, 42).

**FIGURE 6:**
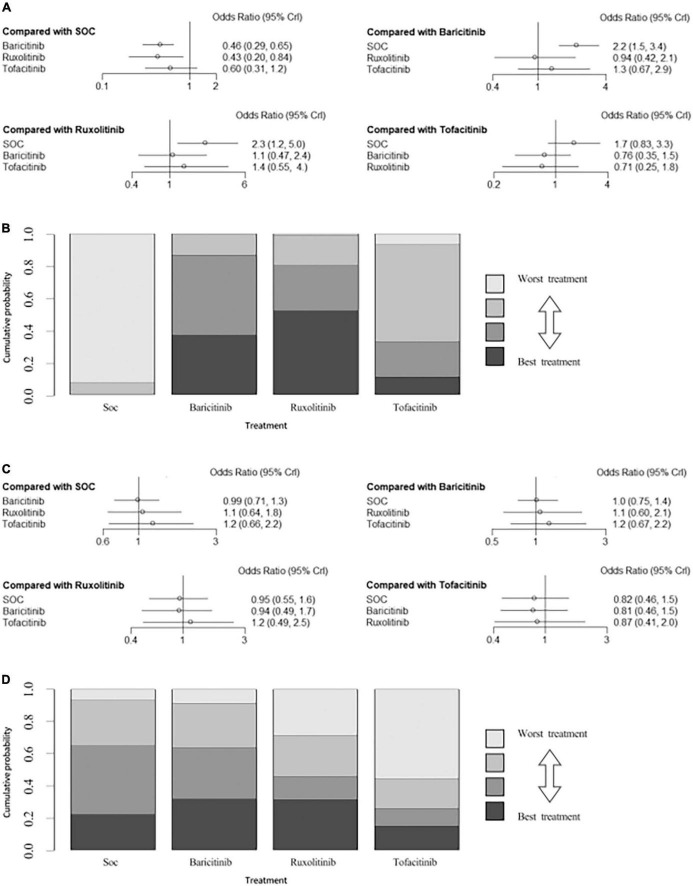
**(A)** Network estimates mortality among JAK inhibitors. **(B)** Rank probabilities among JAK inhibitors for mortality based on the network meta-analysis. **(C)** Network estimates adverse events among JAK inhibitors. **(D)** Rank probabilities among JAK inhibitors for adverse events based on the network meta-analysis. CI, confidence interval.

In individual comparisons for mortality using SOC as the reference, baricitinib, ruxolitinib and tofacitinib were all more likely to reduce the mortality (NMA: OR = 0.46, 95% CI: 0.29–0.65; OR = 0.43, 95% CI: 0.20–0.84; OR = 0.60, 95% CI: 0.31–1.2, respectively; Figure 6A). There were no significant differences among the other comparisons of the NMA for mortality. The assessment of rank probabilities indicated that ruxolitinib (51.5% probability) presented the greatest likelihood of reducing mortality among the evaluated JAK inhibitors, followed by baricitinib (36.9% probability) and then tofacitinib (11.5% probability) (Figure 6B).

#### Adverse events

There were no significant differences among all comparisons of the network meta-analysis for adverse events (Figure 6C). Ranking analysis revealed that baricitinib presented the lowest rate of adverse events (31.7% probability), followed by ruxolitinib (31.3% probability) and then tofacitinib (14.8% probability) (Figure 6D).

#### Two-dimensional plot of primary outcomes

**FIGURE 7:**
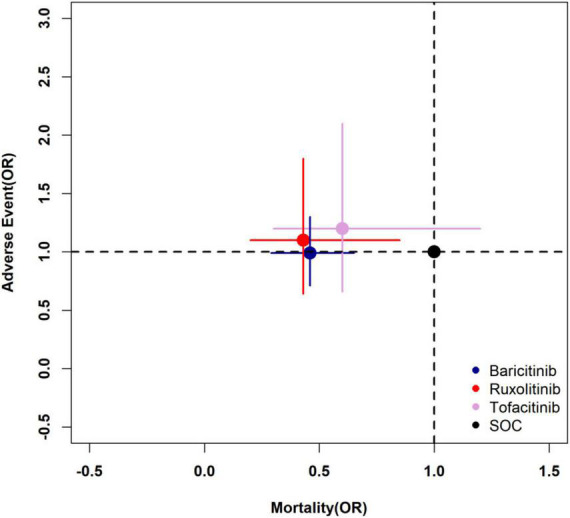
Two-dimensional plot of primary outcomes. The longitudinal coordinate and horizontal ordinate are the odds ratio of mortality and adverse events of each drug to SOC (1.0, 1.0). SOC, standard of care.

The pooled estimates of two primary outcomes were projected to a two-dimensional (2D) plot, thus hierarchical information about efficacy and incidence of adverse events could be obtained simultaneously and help us understand the optimal choice for COVID-19. The 2D plot revealed ruxolitinib as the optimal choice among all JAK inhibitors (Figure 7). Baricitinib could be an alternative option as well.

## Discussion

To the best of our knowledge, this was the first Bayesian network meta-analysis of the efficacy and safety of JAK inhibitors as a potential therapeutic candidate for SARS-CoV-2 specifically. Using JAK inhibitors was associated with a significant reduction in mortality in patients with COVID-19, without clinically meaningful differences in safety from SOC. The present Bayesian network meta-analysis and ranking analysis suggested the best JAK inhibitor in terms of reducing mortality and adverse events for COVID-19 were likely to be ruxolitinib.

In contrast to the four previously published meta-analyses that have summarized published studies of JAK inhibitors (10–13), this research included the latest random control trials and observational studies on the efficacy of JAK inhibitors, and also conducted a systematic pairwise meta-analysis. In the recently published meta-analysis that only included RCTs, the meta-analysis results of mortality were the same as ours, but the subjects of baricitinib included in the study accounted for 97% of the total subjects, so the results of the study were mainly affected by baricitinib. We believe that, as a meta-analysis to discuss the efficacy of JAK inhibitors on COVID-19, the article should increase the research involving other drugs, while ensuring the high quality of the included research. The discussion on safety in this article focused on the occurrence of any grade adverse events or serious adverse events but did not deliberately analyze the occurrence of certain adverse events that deserve attention and were closely related to JAK inhibitors (13). By contrast, our research had a better understanding of treatment-related adverse events in this group of patients.

The safety of JAK inhibitors is a topic of current concern and has significant implications for the treatment of COVID-19. Known adverse events of JAK inhibitors have been described in phase 3 trials with ruxolitinib in myelofibrosis and polycythemia vera (48, 49). Given that JAK2 is essential for the formation of red blood cells and platelets, administration of JAK2 inhibitors induces anemia and low platelet counts. In addition, JAK is involved in immune responses, particularly through interferon-γ, so some infection events, such as hepatitis B infection and reactivation of tuberculosis, are common potential adverse effects (49). Therefore, the thromboembolic and infection risk of JAK inhibitors has been extensively studied and debated since their clinical development (50). In April 2017, the US Food and Drug Administration (FDA) expressed concern about the observed imbalance in thromboembolic events (deep vein thrombosis and pulmonary embolism) in a placebo-controlled clinical trial of baricitinib (51). In September 2021, the FDA issued an updated warning regarding the increased risk of serious heart-related events, cancer, blood clots, and death in JAK inhibitors. As mentioned above, based on the included studies, this study focused on the two most common adverse events, namely thrombotic events and infection. In the study, we found no statistical difference in the incidence of adverse events between the above three JAK inhibitors and SOC. We assumed that it might be related to the shorter course of treatment of JAK inhibitors for COVID-19. As the above FDA warning on adverse events of JAK inhibitors, the drug is usually treated for more than 24 weeks. The warming is based on long-term (up to 72 months) safety data of tofacitinib in patients with rheumatoid arthritis (52). Nevertheless, in the relevant studies we included, most JAK inhibitors have been used only for 7–14 days (53).

Additionally, the mortality and side effects of different JAK inhibitors were compared, specifically in depth by using Bayesian network meta-analysis, which provided a more specific reference for the clinical treatment of COVID-19 patients. Without adding other types of treatment to interfere with the results in this NMA, ruxolitinib is the most likely best treatment, which could significantly reduce all-cause mortality in patients with COVID-19, with no difference in the incidence of adverse events compared with SOC. Although nezulcitinib has also been tried to treat this type of patient, there was only one relevant study and a small number of included people. After the study was included in the NMA, the sensitivity analysis showed that it had a great impact on the original NMA results: the assessment of rank probabilities of the NMA showed that the probability of nezulcitinib being the best treatment was 82.9%, ruxolitinib was 9.52%, baricitinib was 5.74%, and tofacitinib was 1.86% (Supplementary Figure 3), while the rank probabilities assessment results of the original NMA showed that the probability of ruxolitinib being the best treatment was 51.5%, baricitinib was 36.9%, and tofacitinib was 11.5%. Therefore, nezulcitinib was not included in this NMA analysis.

Besides, by comparing the basic characteristics of the included studies, we found that dose of most of the same drugs was consistent. Except for baricitinib, there were four studies (25, 26, 37, 42) that did not provide useful data on dose and one study (29) that used ruxolitinib 5–10 mg daily, which is different from the 10 mg twice a day used in other studies. Therefore, the sensitivity analysis was conducted after the above studies were excluded, and NMA results showed that there was no significant difference between the results before and after exclusion. In terms of efficacy, ruxolitinib still had the largest possibility of the best treatment (58.9% probability) (Supplementary Figure 4), while in terms of safety, three JAK inhibitors had no statistical difference with SOC, and baricitinib was still the best (37.9% probability) (Supplementary Figure 5).

A comprehensive evaluation of different drugs should be made by combining efficacy and safety. In Figure 7, the longitudinal coordinate and horizontal ordinate are odds ratio of mortality and adverse events. Compared with SOC, the treatment with the coordinate point closer to the lower left quadrant in the figure is more likely to be the best treatment. It is true that ruxolitinib and baricitinib are obviously better than tofacitinib in terms of efficacy, but the comparison between them is not obvious. Similar conclusions can also be found through the Bayesian network meta-analysis rank possibility assessment. Among the three drugs, ruxolitinib is the most likely one (51.5%), baricitinib is the second most likely (36.9%), and tofacitinib is the least likely (11.5%). At the same time, it can be found in Figure 7 that there is no significant difference between the three JAK inhibitors and SOC in causing drug-related adverse events in patients. Therefore, based on the comparison between the efficacy and safety of the three drugs, we believe that ruxolitinib is most likely to be the best treatment, while baricitinib can be used in clinical practice as an alternative to ruxolitinib.

The experience with severe SARS-CoV-1 points to a major feature of these infections namely delayed cytokine storm after initial induction and insufficient type I interferon (IFN-I) action (54). The same situation is observed in SARS-CoV-2. During SARS-CoV-2 infection, it induces biphasic disease, first, a flu-like stage, then pulmonary and systemic disease, followed by a potentially suppressed or delayed innate immune response, followed by an emergency signal that may be triggered by sustained viral replication. This could eventually lead to cytokine storms that lead to severe evolution of COVID-19, possibly leading to acute respiratory distress syndrome (ARDS) (13). JAK inhibitors have recently been used to treat inflammatory diseases, such as moderate to severe rheumatoid arthritis and psoriatic arthritis. These disorders are characterized by abnormal activation of the JAK-STAT signaling pathway, which plays a key role in the coordination of immune system responses (55). In addition, JAK/STAT signaling cascades are involved in the control of cell proliferation and survival (56). Therefore, JAK inhibitors hold the potential to cut - off pathological reactions in COVID-19.

Compared with other COVID-19 treatment candidates, all JAK inhibitors have the advantage of being taken orally or inhaled directly (nezulcitinib) and have favorable pharmacokinetic characteristics: short half-life; low plasma protein binding, and minimal interference with Cytochrome P450-mediated biotransformation pathways. JAK inhibitors are mostly eliminated by the kidneys as complete drugs, and partial or slight liver metabolism occurs (57).

However, different JAK inhibitors still have different mechanisms. Ruxolitinib inhibits JAK1 and JAK2 by terminating kinase activity, thus preventing STAT activation and nuclear translocation. Baricitinib is a kinase inhibitor that competes with adenosine triphosphate to efficiently and reversibly inhibit JAK1 and JAK2. Tofacitinib inhibits mainly JAK1 and JAK3 and to a lesser range the JAK2. The inhibition of JAK, especially JAK1 and JAK3, can block the signaling of multiple interleukins, thus reducing inflammatory cascade (55). The differences in the mechanisms of the three drugs may be the main reason for the differences in clinical outcomes. Besides, it has been reported in the previous literature that among the JAK inhibitors, baricitinib shows a particularly high affinity for AP-2 Associated Kinase 1 (AAK1) and is the only drug that can effectively inhibit AAK1 and Cyclin G-Associated Kinase at therapeutic concentrations. Ruxolitinib also shows a relatively high affinity for AKK1, but only tofacitinib does not show significant inhibition on AAK1 (58).

At present, the timing of JAK inhibitor use is also considered to be the key to the success of treatment. Winthrop have proposed a clinical staging system consisting of three COVID-19 stages in terms of illness development (59). The third stage is marked by severe extrapulmonary systemic hyperinflammation syndrome, ARDS, systemic inflammatory response syndrome, and impending multi-organ failure. It is believed that JAK inhibitors should be considered before multi-organ dysfunction. However, there is still no standard for the classification of disease development. Most of the included studies involved patients classified as moderate or severe according to the oxygenation index. The previous relevant meta-analysis also included subgroups classified by other patient severity scores. Current studies are insufficient to perform a network meta-analysis of study outcomes in patients with different disease severity receiving JAK inhibitors. Perhaps there will be differences in the efficacy of different drugs in patients of different severity.

There are some limitations to this research. First, to ensure the integrity of the included studies, in addition to RCTs, this study also included relevant high-quality observational studies. Although the evidence value of such observational studies is low, it is believed that the observational studies that have passed the screening nonetheless have high reference values. Second, the current JAK inhibitor-related network evidence map shows that the baricitinib-related studies account for the majority of all JAK inhibitors studies (14/28), hence also the majority of subjects were treated with that drug. However, because the data leading to the conclusion of better efficacy with ruxolitinib were drawn from relatively few studies, which may have affected the results of this network analysis, more high-quality RCTs on ruxolitinib are looked forward to. Finally, our analysis compares only the efficacy of the drug components but was not designed to consider specific drug doses. At present, most of JAK inhibitors used to treat COVID-19 use their own conventional treatment doses. We hoped that relevant RCT results can be published in the future. In addition, we have collected the ongoing clinical studies of related drugs (Supplementary Table 1), which will be tracked by us continuously.

## Conclusion

JAK inhibitors appear to be a promising treatment for reducing mortality from COVID-19 and do not appear to increase the risk of adverse events. This network meta-analysis suggests that mortality benefits are associated with JAK inhibitors, and among these, ruxolitinib presents the greatest likelihood of having benefits for mortality and adverse events.

## Data availability statement

The original contributions presented in this study are included in the article/Supplementary material, further inquiries can be directed to the corresponding authors.

## Author contributions

RC, LZ, and LG contributed to determining the outline and content of the pairwise and Bayesian network meta-analysis. JN and ZL contributed to retrieving literature. JN, ZL, and ZH contributed to writing a draft of this manuscript. All authors contributed to revising the draft critically for important intellectual content, providing final confirmation of the revised version, being responsible for all aspects of the work, and read and approved the final manuscript.

## Footnotes

1
http://www.prisma-statement.org

## Abbreviations

JAK: Janus kinases
COVID-19: coronavirus disease-2019
SARS-CoV: severe acute respiratory syndrome-coronavirus
CRS: cytokine release syndrome
IL: interleukin
STAT: signal transducer and activator of transcription
SOC: standard of care
NMA: network meta-analysis
PRISMA: Preferred Reporting Items for meta-analysis
NOS: Newcastle–Ottawa Scale
RCTs: Randomized clinical trials
OR: odds ratio
2D: two-dimensional
FDA: Food and Drug Administration
IFN-I: type I interferon (IFN-I)
ARDS: acute respiratory distress syndrome.
