# Supplementary material for: Janus kinases inhibitors for coronavirus disease-2019: A pairwise and Bayesian network meta-analysis
Source: Front Med (Lausanne). 2022 Nov 23;9:973688. doi: 10.3389/fmed.2022.973688 (PMC9727257; doi:10.3389/fmed.2022.973688)
Supplement: Supplementary file 1 [file Data_Sheet_1.docx]

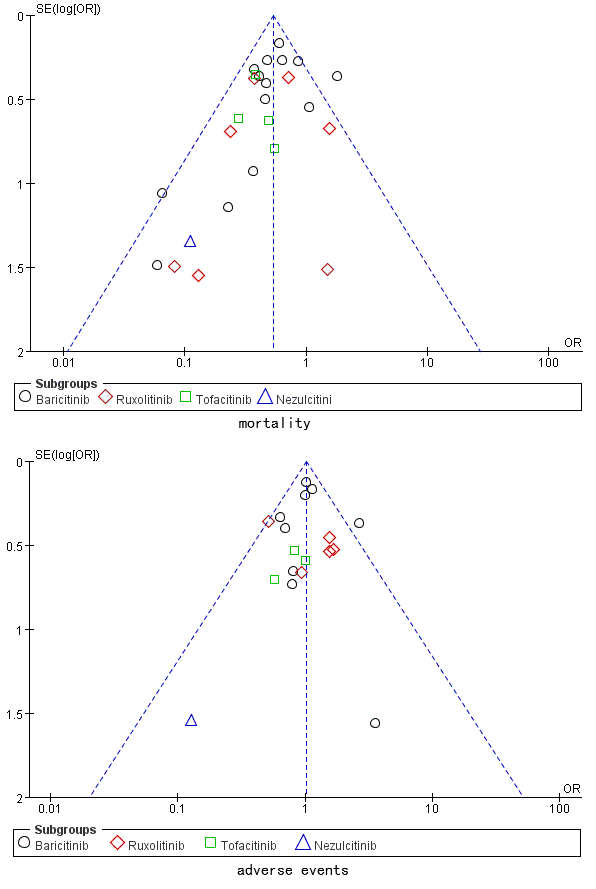


Figure S1: Publication bias of pairwise meta -analysis: mortality (up); adverse events (down)


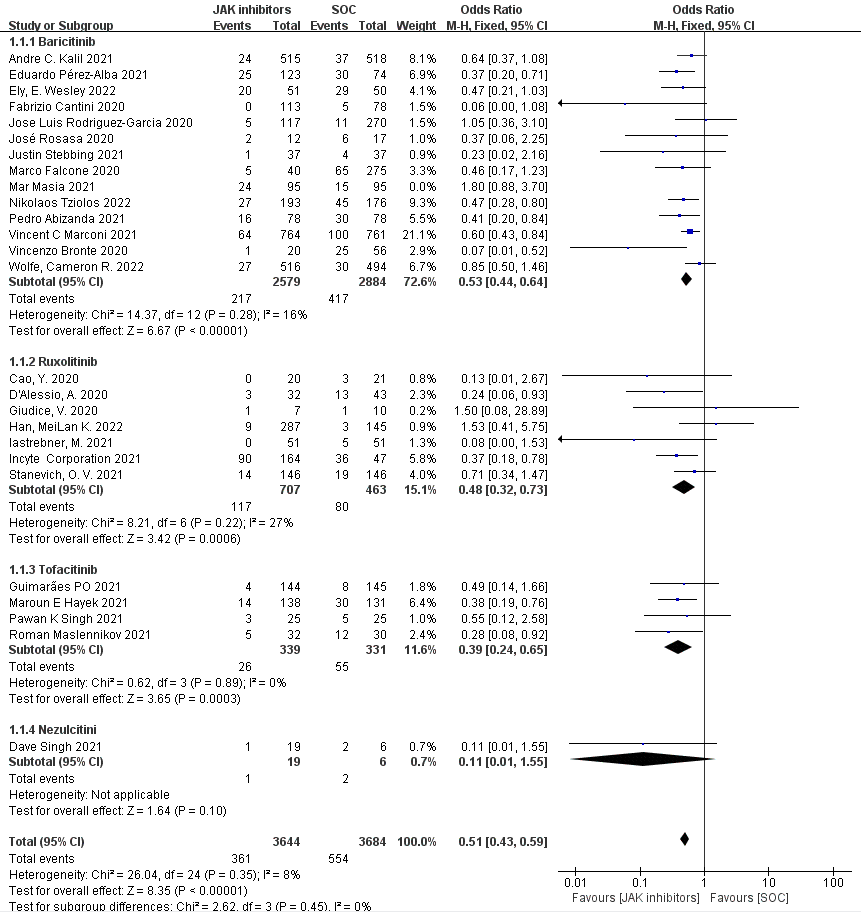


Figure S2: Sensitivity analyses of mortality in pairwise meta -analysis


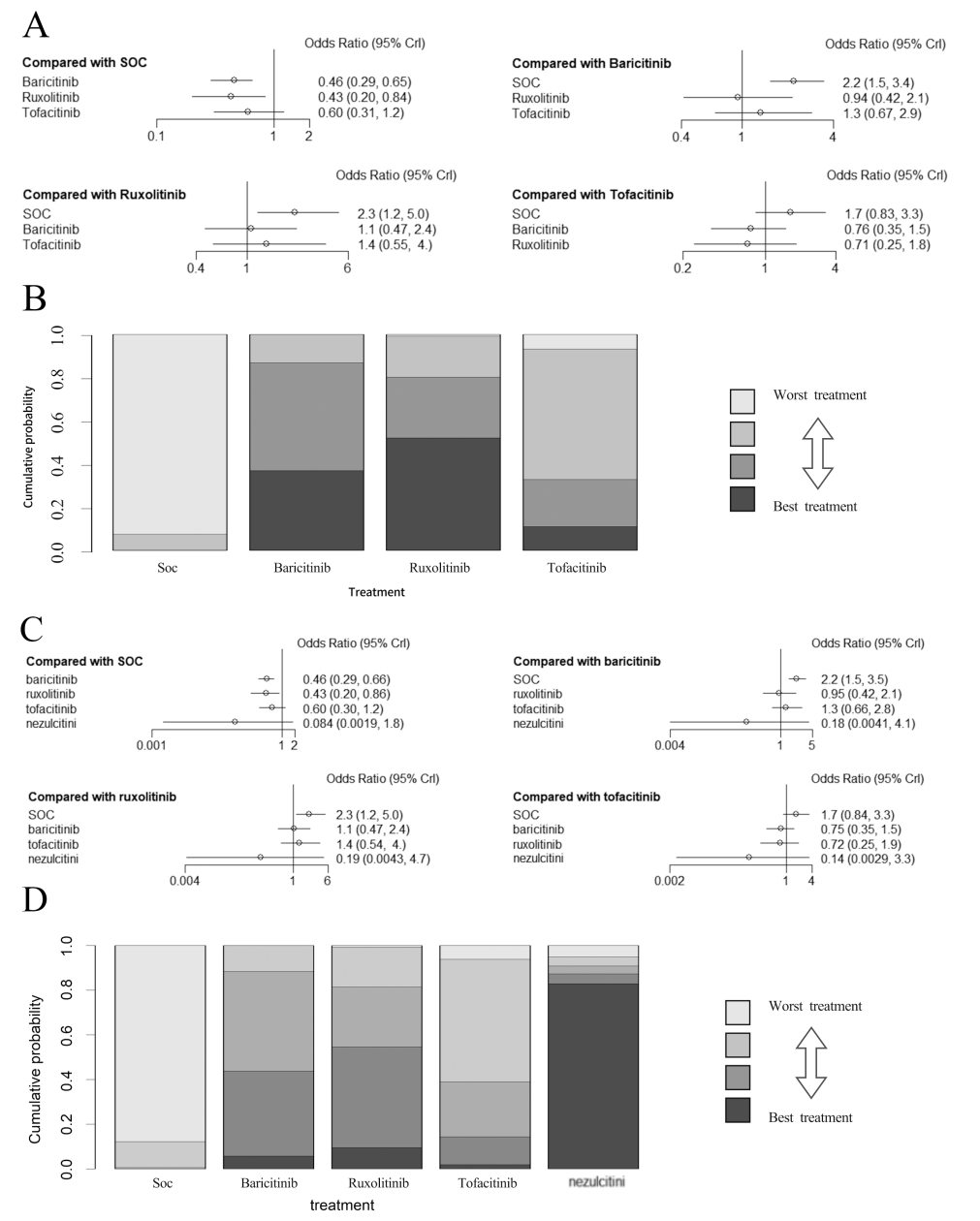


Figure S3: Sensitivity analyses of mortality in network meta -analysis:

S3B. ranking probabilities for each treatment in mortality without nezulcitini;

S3D. ranking probabilities for each treatment in mortality with nezulcitini.


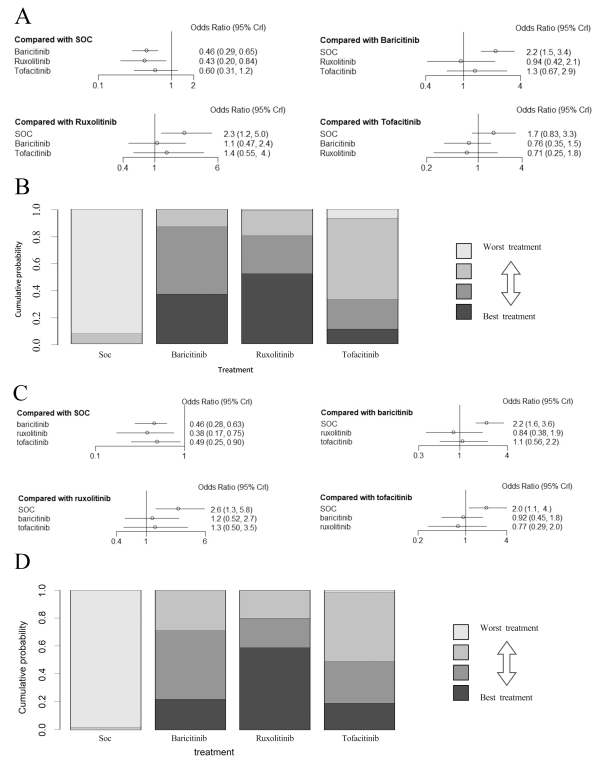


Figure S4: Sensitivity analyses of mortality in network meta -analysis:

S4B. ranking probabilities for each treatment including studies with unclear dose;

S4D. ranking probabilities for each treatment excluding studies with unclear dose.


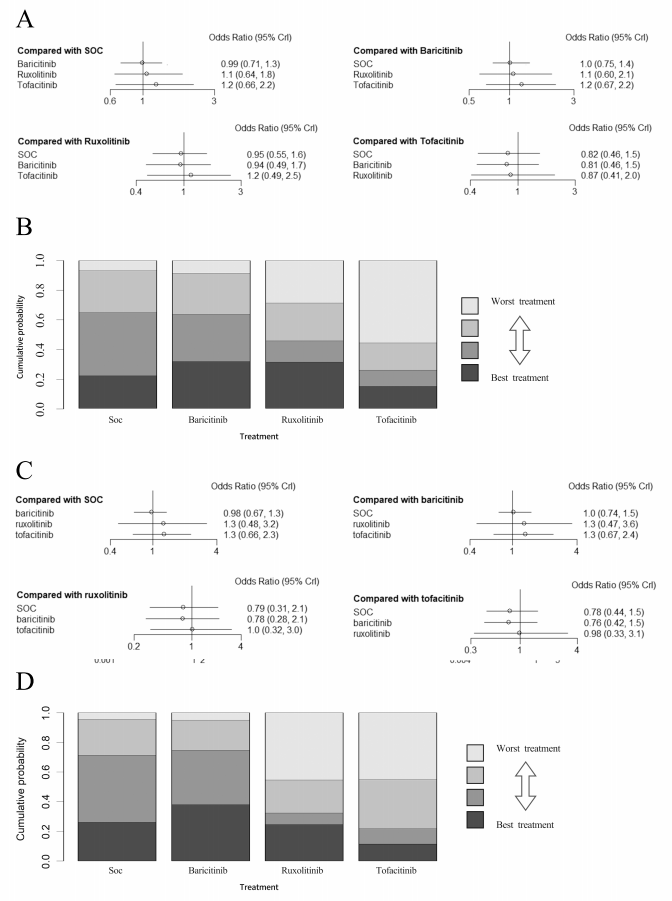


Figure S5: Sensitivity analyses of adverse events in network meta -analysis:

S5B. ranking probabilities for each treatment including studies with unclear dose;

S5D. ranking probabilities for each treatment excluding studies with unclear dose.
